# Biofilms: The Stronghold of *Legionella pneumophila*

**DOI:** 10.3390/ijms141121660

**Published:** 2013-10-31

**Authors:** Mena Abdel-Nour, Carla Duncan, Donald E. Low, Cyril Guyard

**Affiliations:** 1 Ontario Agency for Health Protection and Promotion (OAHPP), Toronto, ON M9P 3T1, Canada; E-Mails: mena_abdelnour@hotmail.com (M.A.-N.); carla.duncan@oahpp.ca (C.D.); 2 Department of Laboratory Medicine and Pathobiology, University of Toronto, Toronto, ON M5S 1A8, Canada; 3 Mount Sinai Hospital, Toronto, ON M5G 1X5, Canada

**Keywords:** *Legionella pneumophila*, legionellosis, amoeba, prozoa, multispecies biofilms

## Abstract

Legionellosis is mostly caused by *Legionella pneumophila* and is defined as a severe respiratory illness with a case fatality rate ranging from 5% to 80%. *L. pneumophila* is ubiquitous in natural and anthropogenic water systems. *L. pneumophila* is transmitted by inhalation of contaminated aerosols produced by a variety of devices. While *L. pneumophila* replicates within environmental protozoa, colonization and persistence in its natural environment are also mediated by biofilm formation and colonization within multispecies microbial communities. There is now evidence that some legionellosis outbreaks are correlated with the presence of biofilms. Thus, preventing biofilm formation appears as one of the strategies to reduce water system contamination. However, we lack information about the chemical and biophysical conditions, as well as the molecular mechanisms that allow the production of biofilms by *L. pneumophila*. Here, we discuss the molecular basis of biofilm formation by *L. pneumophila* and the roles of other microbial species in *L. pneumophila* biofilm colonization. In addition, we discuss the protective roles of biofilms against current *L. pneumophila* sanitation strategies along with the initial data available on the regulation of *L. pneumophila* biofilm formation.

## 1. Introduction

**Figure 1:**
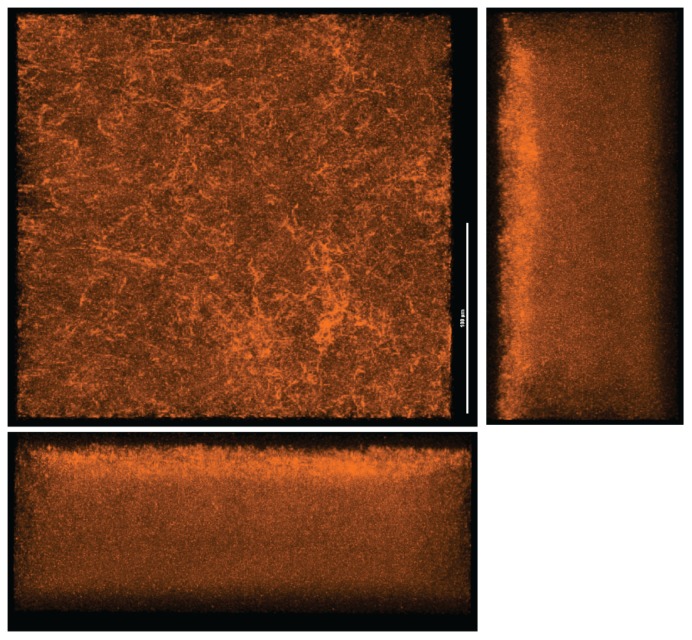
Monospecies biofilm of *L. pneumophila* labelled with the DNA stain Syto62. Scale bar represents 100 μm.

The Gram-negative bacterium *Legionella pneumophila* is responsible for the majority of legionellosis cases and is a significant contributor of community acquired, and hospital acquired pneumonia with a case fatality rate ranging from 5% to 80% [1,2]. *L. pneumophila* is an aquatic pathogen that is ubiquitously found in nature, in both anthropogenic structures and in environmental waters [3–7]. *In vitro*, *L. pneumophila* is able to produce monospecies biofilms (Figure 1) that is believed to contain an extracellular matrix [8,9]. In the environment, *L. pneumophila* can be found in several different habitats, including multispecies biofilms. In naturally occurring multispecies biofilms, the colonization with *L. pneumophila* can be influenced by several other species of microorganisms [10,11]. Of these microorganisms, protozoa are arguably one of the most important in determining *L. pneumophila* persistence, as the pathogen uses protozoa to replicate intracellularly [12]. Co-evolution with multiple species of protozoa has resulted in the development of mechanisms that allow *L. pneumophila* to occupy a very broad host range, and to infect human cells [13–15].

Recent reports suggest that the growth of *Legionella* in biofilms may lead to enhanced virulence. *L. pneumophila* isolates from serogroups 1, 10, and 12 that were collected from biofilms were more cytotoxic towards amoeba than reference outbreak and worldwide epidemic strains [16]. Moreover, initial data suggest that biofilm-derived *Legionella pneumophila* evades the innate immune response in macrophages [17]. As legionellosis is not transmitted from person to person, insights into the ecology of *L. pneumophila* may yield information that can be used to prevent the colonization of anthropogenic systems by *L. pneumophila*. In light of recent discoveries, this review intends to provide an overview of the findings on biofilm production and colonization by *L. pneumophila*.

## 2. Protozoa and *L. pneumophila* Biofilm Formation

**Figure 2:**
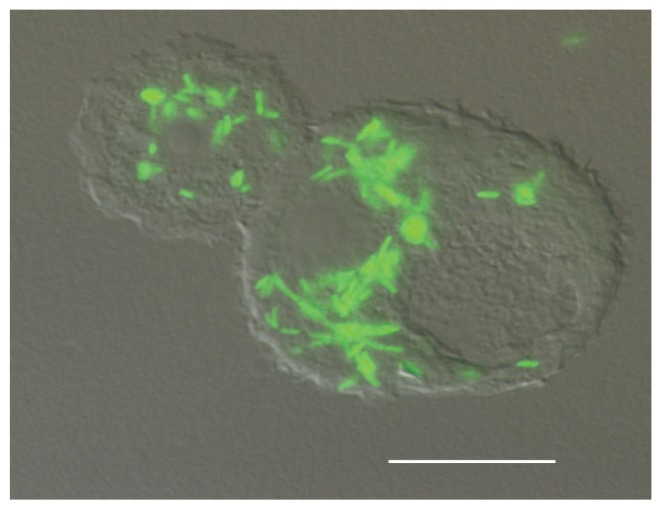
*A. castellanii* infected with *L. pneumophila* expressing green fluorescent protein. Scale bar represents 10 μm.

Protozoa play a crucial role in the lifecycle of *Legionella* species as they provide a habitat for their environmental survival and replication [12,18–21]. In biofilm communities, several amoeba species have been found associated with *L. pneumophila* [22]. To feed, protozoan species often graze on bacteria present in multispecies biofilms, a phenomenon that *L. pneumophila* exploits in order to replicate (Figure 2) [23,24]. As a consequence, the presence of protozoa in anthropogenic water sources has been deemed a risk factor for *L. pneumophila* outbreaks [23]. In fact, the amount of *L. pneumophila* in biofilms is directly correlated with the biomass of protozoa [25]. This is in accordance with *in vitro* models showing that the presence of amoeba species promotes the biofilm formation of *L. pneumophila* on pins of “inverse” microtiter plates [26]. *L. pneumophila* is also capable of growing off the debris from dead amoebae, thus, amoeba may also encourage the replication of *L. pneumophila* indirectly [27]. Floating biofilms, also contain protozoa in association with *L. pneumophila* suggesting that *L. pneumophila* may attach to protozoa in floating biofilms in the absence of available abiotic surfaces [28,29]. In addition to the role of protozoa as a means of replication, the intracellular stage of *L. pneumophila* provides protection from environmental stressors [30,31] including biocides used to disinfect water systems [32,33]. Indeed, biofilms produced with *L. pneumophila* in the presence of thermotolerant amoebae allow *L. pneumophila* to persist after heat treatment [34], demonstrating that amoebae can provide a protective niche for *L. pneumophila* [30].

## 3. Physiochemical Determinants in *L. pneumophila* Biofilm Formation and Colonization

**Figure 3:**
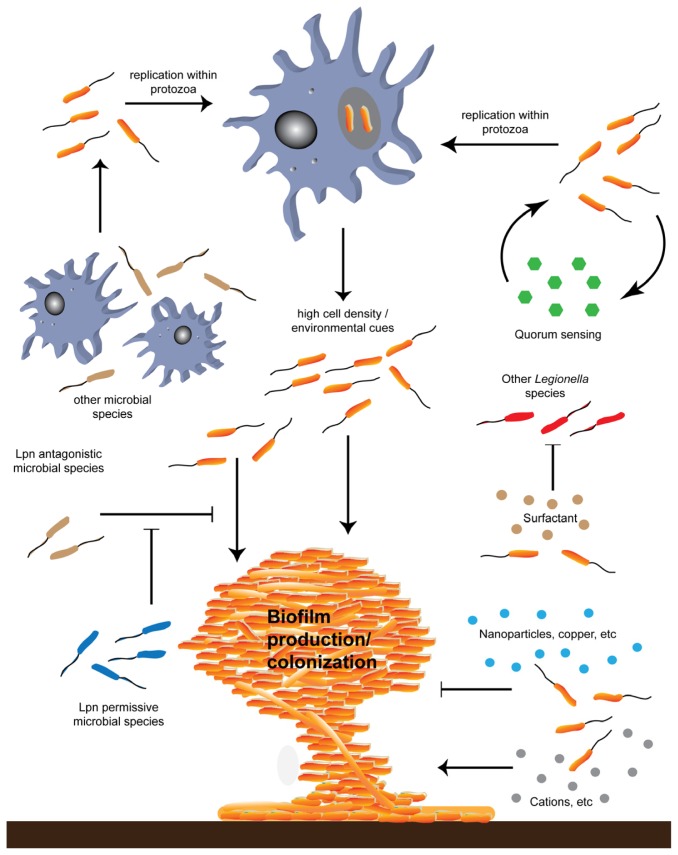
*L. pneumophila* (denoted by Lpn and shown in orange) replicate within environmental protozoa. Uptake within protozoan hosts is promoted by the presence of other amoebae and bacterial species such as *P. aeruginosa* (**top left**) and by environmental cues such as quorum sensing (green hexagons, **top right**). Environmental cues can also influence changes in *L. pneumophila* cell metabolism that favour biofilm production and colonization, which may occur following replication within protozoa or independently of protozoa infection (**middle**). Other microbial species such as *P. aeruginosa* (Lpn antagonistic microbial species, brown) can inhibit *L. pneumophila* colonization (**bottom left**). The presence of other microorganisms such as *K. pneumoniae* alleviates the inhibitory effect of *P. aeruginosa* (Lpn permissive microbial species, blue) and allows *L. pneumophila* to be incorporated within biofilms. *L. pneumophila* produces a surfactant (brown circles), which is toxic to other *Legionella* species (red), and may therefore prevent incorporation of these bacteria within biofilms. Physio-chemical parameters such as divalent cations (grey circles) can favour *L. pneumophila* biofilm colonization while other factors such as the presence of nanoparticles and copper (blue circles) can hinder *L. pneumophila* colonization.

Attachment is the process of one object adhering to another, and when producing surface associated biofilms, attachment of *L. pneumophila* serves as an initial crucial step, whether it is on biotic or abiotic surfaces. Once bacteria are attached to a substratum and a biofilm is formed, the process of spreading and persisting within a new area is defined as colonization. Although *L. pneumophila* can often be found attached to various surfaces in the environment, colonization of existing biofilms in addition to attachment to abiotic substrates is determined by a wide variety of parameters (Figure 3) [35]. One important factor that governs the adherence of *L. pneumophila* in anthropogenic water systems is the composition of the surface material to which the bacteria are adhering [36]. *L. pneumophila* can adhere well to several different plastics that are commonly used in plumbing, whereas copper inhibits its attachment [36–38]. It remains unclear however whether this is due to differences in surface and *L. pneumophila* interactions or because different plumbing materials select for different pioneering species, which establishes the initial biofilm and *L. pneumophila* colonizes afterwards.

Cations are implicated in the attachment of bacteria to different substrata, and can contribute to biofouling [39]. Similarly, both calcium and magnesium were demonstrated to facilitate the attachment of *L. pneumophila* to abiotic surfaces [40]. Elevated zinc, magnesium, and manganese levels are correlated with increased *L. pneumophila* contamination and zinc increases the ability of *L. pneumophila* to bind to host cells such as human lung epithelial cells, suggesting that cations may increase the attachment of *L. pneumophila* to biotic surfaces in addition to abiotic substrata [41–43]. Interestingly as it pertains to the cation dependent attachment of *L. pneumophila*, an orthologue of the *Pseudomonas fluorescens* calcium-dependent cyclic-diGMP regulated protease LapG was identified in *L. pneumophila.* LapG regulates biofilm formation of *Pseudomonas fluorescens* by cleaving the surface adhesin LapA required for biofilm formation [44,45].

In addition to the presence of cations, the availability of carbon favours the colonization of biofilms with *L. pneumophila*, presumably because it provides nutrients for the bacteria to replicate [46]. Notably, the increase in biofilm production due to organic-carbon has only been reported at 20 °C, suggesting that carbon may only influence biofilm production at certain temperatures [47]. Temperature is also an important determinant for *L. pneumophila* biofilm colonization. Studies have shown that heating water above 55 °C can reduce the detectable amount of *L. pneumophila* in water systems, even in the presence of organic carbon sources, however this may be due to a decrease in other biofilm species which may serve as a platform for *L. pneumophila* colonization [48,49].

Static and flow conditions of water play important roles in the biofilm formation and biofilm colonization with *L. pneumophila* in water systems. Stagnation of water in distribution systems seems to favour colonization with *L. pneumophila* [50]. Moreover, Legionnaires’ disease cases have been linked to stagnant water in hospital settings [51]. In accordance with these data, a constant flow in anthropogenic water can decrease the presence of *L. pneumophila* through the use of Venturi systems by preventing the attachment of the bacteria to surfaces [52]. However, biofilms in aquatic environments can persist under turbulent flow conditions [53,54] and maintain a population of *L. pneumophila* [55]. To explain the persistence of *L. pneumophila* under turbulent flow, it was proposed that the bacteria can localize to the sediment where it is less affected by turbulence [56,57]. The settling of *L. pneumophila* in environmental sediments, which was recently linked to quorum sensing, will be discussed below [58,59].

Although the majority of *L. pneumophila* biofilm formation/colonization research has been focused on determining the physiochemical parameters that allow *L. pneumophila* to colonize and form biofilms, little is known regarding the *L. pneumophila* molecular factors that contribute directly to this process. The *Legionella* collagen-like protein (Lcl) was initially identified as an adhesin required for infection of protozoa and macrophages [60]. Subsequently, Lcl was found to be an important mediator of *L. pneumophila* biofilm formation [61]. Lcl facilitates biofilm production by promoting attachment to abiotic substrates as well as cell-cell/cell-matrix interactions [62]. Type IV pili was also implicated in *L. pneumophila* biofilm colonization based in its role in adherence to protozoan cells [63]. However, a site directed type IV pili mutant was shown to colonize biofilms of other organisms as well as wild-type bacteria [64]. In addition to surface exposed adhesins, the twin arginine transport (Tat) secretion system has also been implicated in biofilm formation. Deletion of the *tatB* and *tatC* genes resulted in a significant reduction in biofilm formation, however the specific role that this secretion system plays is unknown [65].

## 4. Regulation of *L. pneumophila* Endogenous Factors that May Influence Biofilm Colonization

For *L. pneumophila*, as well as for other microorganisms, biofilm formation is an environmental response that can promote survival. *L. pneumophila*, like many other microorganisms, responds to environmental cues, which can greatly influence biofilm formation and or colonization (Figure 3). One important environmental prompt is iron, which has important roles in the growth of many organisms, and can influence *L. pneumophila* replication [66]. The addition of lactoferrin, an iron chelator, can directly kill *L. pneumophila* demonstrating the importance of iron in *L. pneumophila* viability [67]. Furthermore, bacterial ferrous iron transport promotes the intracellular replication of *L. pneumophila* in protozoa, which may influence multispecies biofilm colonization [68]. Iron is also required for the production of melanin and it is believed that deletion of the *lbtA* and *lbtB* genes, which encode iron siderophores prevent growth within aquatic biofilms [66]. Interestingly, although iron is essential for biofilm formation, high iron concentrations can inhibit biofilm formation, yet to date the reasons for this are unknown [8].

The ability of bacteria to monitor and respond to cell density is known as quorum sensing and it is a crucial process during biofilm production. Among quorum sensing molecules, α-hydroxy ketones (AHKs) have been identified in *L. pneumophila*, and are similar to the AHKs produced by *Vibrio cholera* [69,70]. Although there is currently no evidence that AHKs regulate *L. pneumophila* biofilm production directly, these molecules regulate a wide variety of traits that may influence *L. pneumophila* biofilm production/colonization indirectly, including virulence (infection of protozoan hosts in multispecies biofilms), extracellular filament production, and sedimentation through the *lqs* gene cluster, which encodes for the AHK synthase LqsA, the AHK sensor LqsS and the response regulator LqsR [58,71,72]. In addition to the products of this gene cluster, an orphan sensor kinase named LqsT regulates competence, a process that is correlated to biofilm formation in other species [59].

The second-messenger molecule cyclic di-GMP (c-di-GMP) is also an important signaling system that allows bacteria to respond to environmental changes [73]*L. pneumophila* has 22 predicted genes related to c-di-GMP production, degradation and/or recognition [74]. One of these genes, *lpg1057*, was found to encode an enzyme responsible for the production of cyclic di-GMP which promotes biofilm formation, and is the only c-di-GMP related gene to date found to directly influence monospecies biofilm production of *L. pneumophila* [75]. In response to amino acid starvation, the alarmone guanosine tetraphosphate (ppGpp) can also regulate *L. pneumophila* gene expression [76]. Although the ppGpp system is mainly linked to the regulation of virulence related traits, this system may indirectly affect environmental biofilm colonization by influencing *L. pneumophila*-amoeba interactions. In addition, sensitivity to ppGpp signaling requires the sigma factor RpoS [77]. RpoS in turn influences LqsR expression, suggesting that virulence related traits regulated by AHKs require multiple environmental signals [78]. In parallel with the ppGpp-RpoS regulation of virulence, downstream is the two-component system LetA/LetS [79]. The LetA/LetS system relieves the repression of virulence related genes by the RNA binding protein CsrA [80]. Despite the initially suspected roles of these transcriptional regulators in surface attachment and biofilm formation, none of the mutants lacking *rpoS*, *letA* or *csrA* were affected in biofilm formation in the *L. pneumophila* strain JR32 [26]. Of the known *L. pneumophila* sigma factors, to date only the flagellar sigma factor FliA has been implicated in the regulation of biofilm production and deletion of *fliA* results in a decrease in biofilm formation in JR32, however it is unclear what downstream or upstream factors are involved in this process [26].

Temperature was mentioned above as being an important determinant for biofilm colonization [48,49]. In addition, temperature can regulate the properties of the biofilms produced by *L. pneumophila* [81]. *In vitro*, at 37–42 °C, monospecies biofilms are mycelial mat-like and are composed of filamentous bacteria whereas biofilms produced at 25 °C are thinner and made up of rod shaped cells [81]. These findings coincide with other studies demonstrating that the filamentation of *L. pneumophila* is regulated by temperature [82]. Filamentous growth occurs in other bacterial species to increase fitness against adverse environmental conditions [83]. In turn, intracellular filamentatous *L. pneumophila* can produce progeny more efficiently than short rod forms [84]. Furthermore, the length of *L. pneumophila* cells has been linked to ppGpp signalling [80]. *In vitro*, biofilms produced at 37 °C are much more robust than at 25 °C [26], and interestingly biofilms produced at 25 °C are more adherent [81]. In addition, the production of the *L. pneumophila* type II secretion system, and type IV pili are temperature regulated, and may influence attachment at different temperatures [85].

## 5. The Role of Non-Protozoa Microbial Species in *L. pneumophila* Biofilm Colonization

Environmental biofilms often contain several different bacterial species [86]. These bacterial species may promote the persistence of *L. pneumophila* in biofilms, while other species inhibit *L. pneumophila*’s colonization (Figure 3). For example, *Flavobacterum breve* and cyanobacterial species can promote *L. pneumophila* growth and colonization in biofilms by providing a source of nutrients [87,88]. *In vitro*, the growth of *L. pneumophila* is necrotrophic when heat killed *Pseudomonas putida* bacteria are given as a nutrient source, however heat killed Gram-positive organisms such *Bacillus subtilis* and *Lactobacillus plantarum* did not alter the growth of *L. pneumophila*, suggesting that *L. pneumophila* is capable of replicating without the presence of protozoan species, and that necrotrophic growth of *L. pneumophila* is restricted to certain microbial species [27].

Summer seasons, which coincide with legionellosis outbreaks, favour the proliferation of *L. pneumophila* in cooling tower microbial populations while other *Legionella* species decrease in number [89]. Based on this shift in abundance it has been hypothesized that *L. pneumophila* may inhibit the growth of other *Legionella* species. In fact, *L. pneumophila* produces a surfactant secreted by the protein TolC, which is toxic to other *Legionella* species, but has no effect on *Pseudomonas aeruginosa*, *Klebsiella pneumoniae* and *Listeria monocytogenes* [90]. Therefore it is tempting to speculate that *L. pneumophila* may influence the growth of other *Legionella* species in their natural environment.

One of the most studied bacteria that can influence *L. pneumophila*’s biofilm colonization ability is *P. aeruginosa*. Although there is a body of evidence suggesting that *L. pneumophila* can coexist in biofilms with *P. aeruginosa*, these studies were performed with inoculums from natural environmental sources, which may contain several different bacterial species [11,38]. In contrast to these studies, monospecies biofilms with *P. aeruginosa* were shown to prevent *L. pneumophila* colonization [26,91]. This phenomenon may be mediated by acylhomoserine lactones (AHLs) produced by *P. aeruginosa* as these AHLs not only inhibit the growth of *L. pneumophila* but also its biofilm production [92]. Furthermore, specific AHLs produced by *P. aeruginosa* can downregulate Lcl production, which is essential for biofilm formation in *L. pneumophila* [62]. Interestingly, the *in vitro* inhibition of *L. pneumophila* colonization by *P. aeruginosa* is alleviated if *K. pneumoniae* is present in the produced biofilm [91]. In fact complex multispecies biofilms that contain both *P. aeruginosa* and *K. pneumoniae* are permissive for *L. pneumophila* colonization [28]. The presence of amoeba seems to also effect whether *P. aeruginosa* is antagonistic to *L. pneumophila* colonization, as biofilms which contain both *Acanthamoeba castellanii* and *P. aeruginosa*, increase the uptake of *L. pneumophila* within *A. castellanii*, and the colonization of *L. pneumophila* in biofilms [93].

## 6. The Resistance of *L. pneumophila* Containing Biofilms to Biocides

There is a great interest in improving methods for disinfecting *L. pneumophila* containing biofilms because of the ongoing threat to human health posed by these organisms in anthropogenic water sources. Due to the intracellular lifestyle of *L. pneumophila* within protozoa, however, it is difficult to tease out whether the resistance of *L. pneumophila* in environmental biofilms is due to the biofilm structure, its association with amoeba or both. It is however evident that environmental *L. pneumophila* found in biofilms are extremely resilient to treatment with biocides [94]. *L. pneumophila* exposed to environmental stresses and/or found within biofilms can enter a viable but non-culturable (VBNC) state, and treatment of water systems with biocides can make *L. pneumophila* enter the VBNC state [95]. This property makes the accurate assessment of the contamination levels with *L. pneumophila* cumbersome since it requires the co-culturing of *L. pneumophila* with amoeba to lift the VBNC state [96].

Recently, nanoparticles have been suggested to be powerful tools to prevent *L. pneumophila* biofilm formation, as nanoparticles are able to disrupt *L. pneumophila*-amoebae interactions and biofilm structure [97,98]. Nanoparticles can also effectively clear *L. pneumophila* from mixed species biofilms and appear to be an attractive treatment option for disinfecting anthropogenic water sources [99]. The most common biocides used to control water-borne pathogens are generally chlorine derivatives, and chlorine derivatives are more efficacious than UV for disinfecting *L. pneumophila* [100–102]. Yet chloramine, one of the most potent chlorine derivative biocides, does not completely eradicate *L. pneumophila* from aquatic biofilms [103,104].

The location of the biofilm can also play a role in resistance to disinfection strategies. This is particularly the case for biofilms formed in sediments, which provide protection to *L. pneumophila* from UV radiation [105]. Furthermore, *L. pneumophila* bacteria grown on a solid surface are more resistant to killing by iodine than bacteria grown in broth, suggesting that there are metabolic differences between surface associated and planktonic phase bacteria [106]. This is consistent with data suggesting that sessile and planktonic *L. pneumophila* in biofilms have different gene expression profiles [8].

## 7. Conclusions

*L. pneumophila* is an environmental pathogen, and understanding the ecology of this pathogen can help to determine methods for preventing its environmental dissemination and the transmission of legionellosis. There are a multitude of factors that can influence whether *L. pneumophila* produces biofilms, and likely many more that remain to be uncovered. Although there have been significant advances in the understanding *L. pneumophila* biofilm formation and colonization in the last several years, there is much that remains unknown. The presence of other microbial species, physiochemical parameters, and *L. pneumophila* gene regulation are all factors that could potentially be exploited to prevent colonization of *L. pneumophila* in anthropogenic systems. The physiochemical parameters, which favour biofilm, formation is a topic of great interest. Research has yielded insight into factors, which could potentially limit *L. pneumophila* growth and may be useful for the prevention of legionellosis. One area of increasing interest is the role of other bacterial species in *L. pneumophila* biofilm production, and the mechanism with which certain species promote *L. pneumophila* growth while other species inhibit it. Another question that remains to be answered is to what extent the intracellular lifestyle contributes to *L. pneumophila* biofilm resistance to disinfection *in situ*. Finally, there is still much unknown about the endogenous factors that *L. pneumophila* utilizes to facilitate biofilm formation, such as what *L. pneumophila* quorum sensing systems regulate biofilm formation and what other factors are involved, (for example an extracellular matrix has been found in *L. pneumophila* monospecies biofilms but has yet to be characterized [8]). Ultimately, this research can yield valuable information that can lead to translational research for prevention and protection against *L. pneumophila* infections.
